# Nano-curcumin for the Treatment and Management of Diabetes Mellitus

**DOI:** 10.2174/0115733998400703250729191528

**Published:** 2025-08-21

**Authors:** Bhagavathi Sundaram Sivamaruthi, Chaiyavat Chaiyasut, Kaliyappan Illakiya, Periyanaina Kesika

**Affiliations:** 1 Office of Research Administration, Chiang Mai University, Chiang Mai, 50200, Thailand;; 2 Innovation Center for Holistic Health, Nutraceuticals, and Cosmeceuticals, Faculty of Pharmacy, Chiang Mai University, Chiang Mai, 50200, Thailand;; 3 Department of Microbiology, Karpagam Academy of Higher Education, Coimbatore, 641021, India;; 4 School of Law, Vel Tech Rangarajan Dr. Sagunthala R&D Institute of Science and Technology, Chennai, 600062, India

**Keywords:** Bioactivity, curcumin, *curcuma longa* l, diabetes mellitus, molecular players, nanomedicine

## Abstract

Diabetes mellitus (DM) is a growing global health concern, placing increasing strain on healthcare systems. Curcumin, the primary bioactive compound in *Curcuma longa* (turmeric), has been reported to exhibit several therapeutic effects, including potential benefits for managing DM. However, its clinical use is limited by poor bioavailability. Nanotechnology, particularly nano-curcumin (nCUR), offers a promising solution by enhancing curcumin's delivery and effectiveness. Preclinical and clinical studies suggest that nCUR may help manage DM and its complications by reducing oxidative stress, genotoxicity, and mitochondrial dysfunction. Despite these promising results, the exact molecular mechanisms of nCUR remain unclear, and clinical evidence is still limited. Furthermore, there is a lack of global guidelines regulating the use of nanomaterials in medicine. In summary, while nCUR shows strong potential as a therapeutic option for diabetes, further research is necessary to elucidate its mechanisms, confirm its clinical efficacy and safety, and establish standardized guidelines for its use in healthcare.

## 1 INTRODUCTION

Diabetes mellitus (DM) represents a major and expanding global health challenge, with its prevalence rising at an alarming rate and posing significant threats to public health systems worldwide [1]. Projections suggest that by 2045, the global number of individuals living with diabetes will reach 783.2 million, reflecting notable increases in prevalence: 11.9% in low-income countries, 21.1% in middle-income countries, and 12.2% in high-income countries [2]. This escalating trend underscores the urgent need for comprehensive prevention, management, and policy initiatives to address the disease across diverse socioeconomic contexts.

In managing DM, a combined approach of non-pharmacological interventions (including dietary modifications, fermented foods, herbs, and lifestyle changes) and pharmacological interventions (including insulin and other medications) is crucial for improving patient outcomes. This strategy ensures a balanced treatment plan that helps control the disease and enhances the quality of life for people with DM [3-10]. The phytochemicals are potent complementary therapeutic candidates for preventing and managing DM [11-13].

Current medications for managing T2DM include several drug classes, each with distinct actions, benefits, and drawbacks. Metformin is the most commonly used first-line drug. It helps reduce liver glucose production, doesn’t cause weight gain, and has a low risk of hypoglycemia, but it can cause stomach upset and shouldn’t be used in people with poor kidney function. Sulfonylureas are an affordable and effective option for lowering blood sugar, but they can also cause weight gain and hypoglycemia. Thiazolidinediones improve insulin sensitivity but may lead to weight gain, fluid buildup, heart failure, and bone fractures. Dipeptidyl peptidase-4 (DPP-4) inhibitors are weight-neutral and have a low risk of hypoglycemia but offer moderate effectiveness and may cause joint pain or pancreatitis. Glucagon-like peptide-1 (GLP-1) receptor agonists help lower blood sugar levels, promote weight loss, and support heart health; however, they can cause nausea and must be administered by injection. They’re not recommended for people with a history of certain thyroid cancers.

Sodium-glucose cotransporter-2 (SGLT2) inhibitors help remove sugar through the urine, aiding weight loss and protecting the heart and kidneys. However, they may increase the risk of urinary infections, dehydration, and a rare type of diabetic ketoacidosis. Meglitinides help control blood sugar levels after meals but require multiple daily doses and can cause hypoglycemia and weight gain. Alpha-glucosidase inhibitors slow down carbohydrate digestion, helping reduce after-meal sugar spikes, but often cause gas and bloating. Treatment choices should be tailored to each person's health conditions, lifestyle, and risks [14, 15]. The detailed mechanisms of action, benefits, and limitations of each medication are displayed in Table **1**.

*Curcuma longa* L., known as Turmeric, is a rhizomatous herbaceous perennial plant of the Zingiberaceae family [18]. The plant originated in India and is now widely cultivated in Sri Lanka, China, African countries, and Southeast Asian countries [19, 20]. Over a hundred curcuma species are reported worldwide, and approximately thirty species are cultivated and consumed in Thailand as food additives, cosmetics, and traditional medicines [21]. Curcuminoids and sesquiterpenoids were the major bioactive compounds of *C. longa* [22]. Curcumin, a major bioactive compound of *C. longa*, is an herbal polyphenol of diacryl heptanoids [23, 24]. Curcumin has been used in Oriental cuisine for its color and flavoring. Still, due to its applications in traditional medicine, several studies have been conducted on it to prevent and treat health complications [25, 26]. Curcumin has been utilized as a medicinal compound with various bioactivities, including anticancer, antioxidant, anti-inflammatory, antimicrobial, anti-diabetic, neuroprotective, immune-modulating, anti-COVID-19, and anti-mutagenic properties [27-40].

Nanotechnology is an emerging technique in several fields, including communication [41], electronics [42], environmental science [43], agriculture [44, 45], food science [46], health science and medicine [47-52]. Nano-curcumin (nCUR) improves curcumin's bioavailability. Although curcumin and nCUR have similar chemical structures, studies revealed that nCUR may enhance curcumin's therapeutic effects, including controlled drug release [53-55]. For example, nCUR has more significant effects on serum lipid profiles than free curcumin, possibly due to its high solubility and bioavailability [56].

Curcumin and nCUR have several beneficial effects, including reducing glycemia and blood lipid levels, as observed in several *in vivo* studies using rodent disease models. Its preventive and therapeutic effects are primarily attributed to its antioxidant and anti-inflammatory properties. Despite the observed positive health outcomes, the limited solubility and bioavailability of curcumin continue to pose significant challenges. To overcome these barriers, researchers have developed several strategies, including advanced drug formulations, nano-drug delivery systems, and the creation of curcumin analogs, all aimed at enhancing its absorption and effectiveness [57].

nCUR offers significant advantages over native curcumin, particularly in enhancing bioavailability, stability, and therapeutic efficacy. Curcumin's clinical application is hindered by its poor water solubility, rapid metabolism, and low systemic bioavailability. For instance, even high doses of oral curcumin result in minimal serum concentrations, thereby limiting its therapeutic potential. In contrast, nCUR formulations, typically ranging from 10 to 100 nm, enhance solubility and facilitate improved cellular uptake, resulting in higher plasma concentrations and prolonged circulation times [58, 59].

Moreover, nCUR has demonstrated superior efficacy in preclinical and clinical studies. nCUR outperforms native curcumin in reducing oxidative stress, improving insulin sensitivity, and enhancing liver and pancreatic health [60, 61]. These findings underscore the potential of nCUR as a more effective therapeutic agent for managing diabetes and related metabolic disorders.

Although several reviews on this subject already exist, this manuscript provides a concise overview of recent preclinical and clinical research on the anti-diabetic properties of nCUR, exploring the potential mechanisms underlying these effects. Furthermore, it addresses key challenges and regulatory issues related to nanomaterials and nanomedicine.

The relevant scientific literature has been collected by searching the databases (Scopus, PubMed, Google Scholar) using the keywords “Curcumin + Diabetes,” “Curcumin + Diabetes mellitus,” “Nano Curcumin + Diabetes mellitus,” and “Nano Curcumin + Diabetes.” The relevant scientific studies in English have been selected for the preparation of the manuscript.

## 2 SOME OF THE CONCERNS ABOUT THE NANOMATERIALS

Nanotechnology is regarded as a double-edged sword. One edge highlights potential health benefits, while the other emphasizes the difficulty of regulating nanotechnology due to its complex and varied nature, depending on the application being considered for regulation.

Nanoparticles have shown potential in enhancing the efficacy of drug delivery systems compared to conventional therapies. However, regulatory frameworks remain underdeveloped despite the growing interest in nanomedicine and its expected impact on the pharmaceutical sector. This regulatory gap poses challenges for ensuring legal certainty for manufacturers, policymakers, healthcare professionals, and consumers. The regulatory bodies of various countries, including the National Institutes of Health, the European Science Foundation, and the European Technology Platform, have different opinions on defining nanomaterials. This inconsistency may hinder funding, research, and development, negatively influencing public perception and acceptance of nanomedicine-based products [62]. The barriers to the development of nanomedicines are the physicochemical characterization, compatibility and toxicity, pharmacodynamics and kinetics, scale-up, and good manufacturing practices [63].

The exclusive properties of nanomaterials do not match the existing regulations and fall outside their scope. This gap poses a significant challenge in ensuring the safety of consumers, workers, and the environment. Globally, regulatory authorities face challenges in developing guidelines that strike a balance between innovation and safeguarding public health. The lack of uniform testing methods and the difficulty of interactions at the nanoscale further complicate regulatory decisions. A thorough assessment must be undertaken to ensure the safety of nanoparticles used in medicine and food. Therefore, a robust partnership among scientific researchers, policymakers, and industry stakeholders is necessary to develop comprehensive regulatory frameworks for nanomaterials. Of the regulatory industry's several challenges, the first is the lack of a standardized, clear definition of whether nanomedicines are used in medicine or other applications. Secondly, we don’t know which qualities of nanomedicine are significant for safety and efficacy. Additionally, it is challenging to determine which principles should be applied to regulate or control whether the proposed product can be considered a nano-similar product. Therefore, without understanding these, it is challenging to justify precise nanomedicine regulations [62, 64].

Proper guidelines and protocols for nanoparticles should be developed to minimize exposure and address potential risks and hazards. The safe handling, production, and disposal of nanomaterials are crucial to minimize potential risks [65]. The small size of the nanomaterials, their larger surface area, and exclusive reactivity could lead to unexpected biological interactions and toxicity [66]. Interdisciplinary research that bridges material science, toxicology, and biology is necessary to mitigate the potential risks associated with nanomaterials. The improved human capabilities, privacy concerns in the age of nanoscale sensors, and the equal distribution of benefits all permit thoughtful consideration.

Adopting nanotechnology and advanced materials also advances ethical and societal concerns that must be considered. Ethicists, social scientists, and policymakers should play a crucial role in anticipating and addressing ethical and societal concerns [67]. The regulatory challenges and evaluation processes associated with using nanomaterials in medicine, as well as guidelines for the industry, have been extensively documented [62, 63, 68, 69]. The key issues in regulating nanomaterials are summarized in Fig. (**1**).

## 3 TYPES OF NANO-FORMULATIONS FOR THE DEVELOPMENT OF NCUR

Curcumin has shown promising therapeutic potential. However, its clinical application is limited due to its poor aqueous solubility, rapid metabolism, and low systemic bioavailability. To overcome these challenges, various nano-formulations have been developed, each offering distinct advantages in terms of stability, release profiles, and therapeutic efficacy.

Liposomes are spherical vesicles composed of phospholipid bilayers that can encapsulate curcumin, enhancing its solubility and stability. The curcumin-loaded liposomes exhibit improved cellular uptake and sustained release, leading to enhanced antioxidant activity and potential therapeutic benefits [70, 71].

Nanomicelles are colloidal carriers formed by amphiphilic block copolymers, which can solubilize hydrophobic drugs like curcumin. A study reported that curcumin-loaded nanomicelles significantly enhanced the solubility and oral bioavailability of curcumin [70, 72].

Solid lipid nanoparticles (SLN) are lipid-based carriers that provide controlled release and enhanced stability for curcumin. Research indicates that curcumin-loaded SLNs exhibit improved absorption and long-term stability upon oral administration, with formulations containing PEGylated surfactants showing greater bioavailability [73].

Polymeric nanoparticles, such as those made from polylactic-co-glycolic acid (PLGA), offer controlled drug release and improved bioavailability. PLGA-encapsulated curcumin has higher serum concentrations and extended circulation times compared to free curcumin, enhancing its therapeutic efficacy [74].

Nanogels are three-dimensional, cross-linked polymer networks that can encapsulate curcumin, providing sustained release and improved stability. Curcumin-loaded nanogels have demonstrated enhanced stability and antitumor effects, particularly in breast and pancreatic cancers [70, 75].

Niosomes are non-ionic surfactant-based vesicles that can encapsulate curcumin, enhancing its solubility and stability. The curcumin-loaded niosomes have shown improved pharmacokinetics and therapeutic benefits in preventing oral cancer progression in animal models [76].

Magnetic nanoparticles can be functionalized to deliver curcumin to specific sites under the influence of an external magnetic field, thereby enhancing targeted therapy. The curcumin-loaded magnetic nanoparticles exhibit enhanced cellular uptake and growth inhibition in cancer cells [75].

Gold nanoparticles can be used to deliver curcumin, offering advantages such as biocompatibility and ease of functionalization. The curcumin-loaded gold nanoparticles can enhance the photothermal and radio-sensitizing effects, potentially improving cancer therapy outcomes [75]. The key advantages of each nano formulation are provided in Table **2**.

## 4 PRECLINICAL STUDIES ON NANO-CURCUMIN AND DIABETES MELLITUS

The impact of nano-encapsulated curcumin (nCUR) supplementation on pancreatic islets and beta cells of streptozotocin (STZ)-induced type 1 DM rats was studied. Rats were supplemented with 10 or 50 mg/kg curcumin or nCUR before the STZ treatments. Afterward, rats were treated with 60 mg/kg of STZ, and they were killed after 72 h. Samples were then collected from the pancreas and blood. In toxicity experiments, animals were treated with curcumin (500 mg/kg) or nCUR (25, 50, or 100 mg/kg) for 28 days *via* oral gavage. Blood and organs were collected to determine the histological and hematologic changes. The changes in blood glucose, plasma insulin, and inflammatory cytokines (Interleukin (IL)-1α, IL-1 β, IL-2, IL-4, IL-5, IL-6, IL-10, IL-12p70, IL-13, IL-17A, TNF-α, IFN γ, G-CSF, GM-CSF) levels were measured. Results showed that non-fasting glucose level was reduced by up to 37% in nCUR-treated animals, while CUR reduced only 12% glucose level. Terminal deoxynucleotidyl transferase deoxyuridine triphosphate nick end labeling (TUNEL) assay and hematoxylin and eosin staining revealed that nCUR treatment suppressed the beta cell death. Minimal histiocytic infiltration and reduced inflammation were observed in curcumin and nCUR-treated groups. ROS-induced DNA damage marker (8-oxo-20-deoxyguanosine) was reduced significantly in the pancreas by the nCUR treatment. The toxicity study revealed that the nCUR treatment did not harm the experimental animals. The study claimed that nCUR could be a safe and effective agent for improving beta cell function [77].

The study suggests that satellite glial cells (SGCs) play a crucial role in the development of diabetic neuropathic pain (DNP) and that the purinergic P2Y12 receptor on these cells in the dorsal root ganglia (DRG) is involved in mediating this pain. The research highlights that diabetic neuropathy (DN) is associated with increased expression of P2Y12 receptors on SGCs, leading to enhanced mechanical and thermal hyperalgesia. This hyperalgesia is thought to be driven by the upregulation of pro-inflammatory cytokines, specifically IL-1β and Cx43 (connexin 43), in the DRG of diabetic rats. The study further demonstrates that nCUR (a potential treatment, possibly a specific compound or therapeutic agent) can reduce the expression of IL-1β, Cx43, and p-Akt (phosphorylated Akt, a protein involved in cell signaling) in the DRG of diabetic rats. Furthermore, nCUR treatment also suppressed the activation of SGCs and reduced CGRP (Calcitonin Gene-Related Peptide) expression in DRG neurons, which are often associated with pain and inflammation. These findings suggest that nCUR treatment attenuates pain by decreasing the expression of the P2Y12 receptor on SGCs, reducing mechanical and thermal hyperalgesia in diabetic neuropathy [78].

STZ-induced diabetic rats were treated with nCUR and AGE (Aged garlic aqueous ethanol extract) (300 or 500 mg/kg). AGE-treated group showed an increase in insulin level and body weight. In contrast, it reduced glucose, creatine kinase-isoenzyme (CK-MB), and lactate dehydrogenase (LDH) levels compared to the control group. The levels of CK-MB, LDH, triglycerides (TG), and aspartate aminotransferase (AST) were decreased in the nCUR-treated group. But body weight, insulin, glucose, and total cholesterol levels were not significantly altered. Transcriptional expression of the Mn-SOD gene was observed in the heart tissue of the nCUR and AGE-treated rats. AGE treatment effectively suppressed the expression of the advanced glycation end-product gene (RAGE). The study also revealed that nCUR and AGE treatments restored the structural integrity of the myocytes compared to the untreated control group [79].

Curcumin-loaded polylactide-poly (ethylene glycol) copolymer nanoparticles (CUR-PLA-PEG) or free curcumin were supplemented to the STZ-induced diabetic rat model and assessed for their beneficial effects. Liver inflammation, characterized by increased AST and alanine aminotransferase (ALT) levels, was observed in the DM rats. Additionally, an increase in malondialdehyde (MDA) and nitric oxide, and a decrease in reduced glutathione (GSH), were observed in diabetic rats. The reduced level of peroxisome proliferator-activated receptors (PPAR-γ) and increased expression of nuclear factor kappa B (NF-κB), transforming growth factor-β1, and cyclooxygenase-2 were observed in DM rats. Free curcumin and CUR-PLA-PEG treatment ameliorated the negative changes associated with diabetes, but CUR-PLA-PEG showed more significant positive results. The study claimed that CUR-PLA-PEG mitigates liver inflammation in STZ-induced DM rats [80].

Nicotinamide and STZ-induced T2DM male Wistar rats were treated with free curcumin (100 or 200 mg/kg/day) or nCUR (100 or 200 mg/kg/day) for 28 days, and their impact on insulin resistance, blood sugar level, and lipid profile was studied. Both free curcumin and nCUR treatments reduced fasting blood sugar (FBS), apelin, triglycerides (TG), low-density lipoprotein (LDL), and very-low-density lipoprotein (VLDL) levels in T2DM rats; however, nCUR showed a more significant impact. Also, nCUR treatment significantly improved the high-density lipoprotein (HDL) level compared to other groups. The study revealed that 100 mg/kg/day improves lipid profile effectively [56].

STZ-induced DM male Wistar rats were treated with free curcumin or nano-micelle curcumin (40 or 80 mg/kg of feed/day) for 42 days. Their impact on body and organ weights, insulin resistance, adiponectin, blood sugar levels, and lipid profiles was studied. Nano-micelle curcumin supplementation effectively increased body weight, and heart and pancreas weights in DM rats. Additionally, there was a significant reduction in serum AST, ALT, glucose, TG, cholesterol, and LDL-C (LDL-Cholesterol), as well as an increase in insulin resistance. Conversely, serum levels of insulin, adiponectin, and HDL-C (HDL-Cholesterol) were elevated. At the same time, no significant changes were observed in the free curcumin-supplemented group compared to the DM control. The improvement was dose-dependent; higher doses (80 mg/kg of feed per day) effectively protected against STZ-induced DM. The study claimed that nano-micelle curcumin is a potent complementary supplement to manage DM-associated complications [81].

STZ-induced male T2DM Sprague-Dawley rats were treated with free curcumin or nCUR (100 mg/kg/day) for 1 month and assessed for changes in glucose, insulin, lipid profile, oxidative markers, and pancreatic and duodenal homeobox 1 (PDX1) expression. The immunohistochemistry study was also employed. The results showed that supplementation with free curcumin and nCUR improved glucose and insulin levels. Significant increases and decreases in the number of immunoreactive beta cells in the insulin and caspase-3 expressions were observed. The study suggests that both free curcumin and nCUR have the potential to serve as therapeutic agents for managing DM. However, nCUR demonstrates greater efficacy than free curcumin [82].

STZ-induced T2DM Wistar rats were treated with free curcumin or nCUR (100 or 200 mg/kg/day) for 28 days and evaluated for changes in FBS and BDNF (Brain-derived neurotrophic factor) expression. Free curcumin and nCUR supplementation increased the serum BDNF levels and decreased the FBS, food, and water intake in T2DM rats. It can be confirmed that nCUR (100 mg/kg/day) is more effective than free curcumin in enhancing serum BDNF levels. Furthermore, it is worth noting that the body weight of the intervention groups did not show a significant reduction compared to the DM control group. The study stated that nCUR is a potent antidiabetic therapeutic agent [83].

The succinyl curcumin conjugated chitosan polymer-prodrug nano micelles (SCCPN) supplementation significantly improved the FBS, lipid profile, and carbohydrate metabolizing enzyme in STZ-induced T2DM Balb/C Mice. In detail, SCCPN was developed and studied for its stability, solubility, drug loading capacity, and controlled drug release. The results showed that SCPN is stable and good enough for the controlled release of the drug. 100 mg/kg BW/day of free curcumin, SCCPN, or standard drug (glibenclamide) was supplemented to DM mice for 21 days. The experiments revealed that SCCPN restored the DM-associated damaged metabolic pathway more effectively than free curcumin and standard drugs. Particularly significant levels of reduction in FBS, TG, LDL-C, total cholesterol, alkaline phosphatase, glucose-6-phosphate dehydrogenase, and serum glutamate oxaloacetic transaminase, and an increase in HDL-C were observed in the supplemented group. The study revealed that SCCPN significantly restores the disrupted metabolic pathway in T2DM mice, demonstrating superior efficacy to glibenclamide and free curcumin [84].

Ganugula *et al.* reported that nCUR + long-acting insulin (LAI; Lantus^®^) improves DM-associated kidney disease through the p38/p53 signaling pathway. STZ-induced T2DM leads to kidney basement membrane thickening, tubular atrophy, and impairment of the podocyte cytoskeleton in Sprague-Dawley rats. Free curcumin (40 mg free curcumin/kg BW/day), nCUR (20 mg nCUR), or nCUR + LAI (20 mg nCUR + 2 IU LAI) was supplemented to T2DM rats for 10 weeks and assessed for their impact on kidney health and other biochemical parameters. The results showed that nCUR and nCUR + LAI significantly improved the kidney lesions. This improvement was associated with the nullification of P38 and P53 hyperactivation, and it suppressed the expression of renal NOD-like receptor protein 3, nuclear factor kappa-light-chain-enhancer of activated B cells, caspase 3, mitogen-activated protein kinase 8, and IL-1β, indicating normalization of the inflammasome and apoptotic pathways. The study demonstrated that therapies targeting the reduction of hyperglycemia and inflammation may offer a more effective management of DM and its associated complications [85].

The supplementation of nCUR encapsulated in fenugreek mucilage significantly reduced the fasting blood glucose level (392 to 187 mg/mL) in STZ-induced T2DM Wistar rats [86].

Dexamethasone-induced diabetic rats supplemented with curcumin or nCUR exhibited significantly increased body weight. Insulin resistance and FBS levels were decreased in the curcumin or nCUR-treated group. TG, LDL, MDA, aspartate transaminase, alanine transaminase, and cholesterol levels were reduced in the pancreatic and liver tissues of the curcumin or nCUR-treated group. Also, levels of albumin, HDL, GSH, SOD, and glucose transporter type 4 were increased. The nCUR treatment showed more significant protective effects compared to free curcumin [61]. A recent study reported that the poly(lactide-co-glycolide)-based nCUR effectively delays the onset of diabetes by reducing oxidative stress, DNA damage, and mitochondrial dysfunction [87] (Table **3**).

A biocompatible dressing composed of a curcumin-loaded polyglycerol sebacate/poly lactic acid (CUR-PGS/PLA) hydrogel has been reported to promote wound healing in diabetic rats. The CUR-PGS/PLA-treated group exhibited faster wound healing, thicker new skin, increased blood vessel growth, enhanced collagen production, and higher levels of healing-related proteins, including transforming growth factor-β and vascular endothelial growth factor, compared to the control group. This group also exhibited the lowest levels of inflammation and cell death, along with reduced levels of inflammatory markers, including IL-1β and TNF-α. In conclusion, the curcumin-loaded PGS/PLA hydrogel significantly enhanced wound healing in diabetic rats by promoting tissue repair and reducing inflammation [88].

## 5 FINDINGS OF THE NCUR IMPACT ON THE HEALTH STATUS OF DM HUMAN SUBJECTS

Sotoudeh and colleagues conducted a randomized, double-blinded, placebo-controlled clinical trial (RCT) and investigated the effect of nano-curcumin intake [nano-curcumin capsules (curcumin 72%, desmethoxycurcumin 25%, and bisdemethoxycurcumin 3%); dose of 80 mg daily for 8 weeks] on the severity of diabetic sensorimotor polyneuropathy in diabetic patients. Compared to the control group (*n* = 40, who consumed placebo capsules), the intervention group (n = 40) showed a decrease in hemoglobin A1c (HbA1c), FBS, total neuropathy score, and total reflex score, indicating improvement and reduction of diabetic sensorimotor polyneuropathy in diabetic patients [89]. Sotoudeh and colleagues [90] also studied the effect of nCUR supplements on stress, anxiety, and depression in DM patients with peripheral neuropathy. Intervention of nCUR (nCUR capsules; dose 80 mg daily for 8 weeks) significantly decreased the mean score of anxiety and depression in the patients (*n* = 40) with DM neuropathy compared to that of the placebo group (*n* = 40). Consuming nCUR has no statistically significant effect on the stress score between the intervention and placebo groups. Therefore, the study results state that 8 weeks of supplementation of nCUR shows a beneficial effect in lessening the anxiety and depression scores in diabetic patients with peripheral neuropathy [90].

Shafabakhsh *et al.* [91] investigated the effect of nano-curcumin supplementation on the metabolic status of diabetic patients undergoing hemodialysis in an RCT. The diabetic patients on hemodialysis in the intervention group (*n* = 30) consumed nano-curcumin capsules with a daily dose of 80 mg for 12 weeks. They showed reduced fasting plasma glucose and serum insulin levels compared to the control group, which consisted of 30 DM patients on hemodialysis who received placebo capsules. The intake of nCUR also significantly decreased LDL-C, VLDL-C, the total cholesterol/HDL cholesterol ratio, TG, plasma MDA, and serum high-sensitivity C-reactive protein (hsCRP) in DM patients on hemodialysis compared to the control group. Intake of nano-curcumin capsules also increased the total nitrite levels and plasma total antioxidant capacity in DM patients on hemodialysis compared to the respective metabolic parameters of diabetic patients on hemodialysis who consumed placebo capsules. This study indicated that the nCUR capsules had a health-promoting effect on the metabolic profile in DM patients undergoing hemodialysis [91].

Dastani *et al.* conducted an RCT investigating the effect of combination therapy with nano-curcumin on cardiovascular risk factors in T2DM patients with mild to moderate coronary artery disease, defined as less than 70% stenosis on angiography. The intervention of nCUR with a dose of 80 mg per day along with medications for 90 days significantly alleviated the levels of lipoprotein and hsCRP, and their respective mean percentage of change was reduced in the DM patients (intervention group, *n* = 32) compared to that of the placebo group (*n* = 32). The combination therapy with nCUR decreased the number of patients with severe hsCRP level category, thereby increasing the number of patients with mild and moderate hsCRP level category, indicating that nCUR might prevent the progression of atherosclerosis and reduce the inflammation index in terms of declining the levels of hsCRP, and cardiovascular events in T2DM patients with coronary artery disease [92].

Asghari *et al.* reported the beneficial effect of nCUR (Dose 80 mg, 1 capsule a day) and two omega-3 polyunsaturated fatty acids, namely eicosapentaenoic acid (EPA) and docosahexaenoic acid (DHA) (daily intake of 2 capsules of High EPAquatic^®^ omega 3 supplements; each 1000 mg of High EPAquatic^®^ capsule containing 500 mg of EPA and 200 mg of DHA) on the various health indicators such as anthropometric, glucose homeostasis, and gene expression markers of cardio-metabolic risk in patients with T2DM. Twelve weeks of intervention with EPA and DHA, along

 with nCUR, resulted in a significant decrease in blood insulin and hsCRP levels. They also significantly increased the total antioxidant capacity level in patients with T2DM compared to the placebo group. The nCUR group demonstrated a significant reduction in LDL levels in diabetic patients compared to the control group and the EPA + DHA group. The serum cholesterol levels in the DM patients were significantly reduced in the EPA + DHA group compared to the nCUR group and the control group. The DM patients showed decreased serum vascular endothelial growth factor levels, which were significantly different between the EPA+DHA group and the nCUR group, between the EPA + DHA group and the placebo group, between the EPA + DHA + nCUR group and the nCUR group, and between the EPA + DHA + nCUR group and the placebo group. Significant differences were not shown in the levels of FBS, HbA1C, Quantitative Insulin Sensitivity Check Index (QUICKI), and Homeostatic Model Assessment of Insulin Resistance (HOMA-IR) index between the four groups (EPA + DHA group, nCUR group, EPA + DHA + nCUR group, and control group). The study results indicate that a 12-week intervention of EPA and DHA, along with nCUR, might show a beneficial effect on oxidative stress, inflammation, and metabolic parameters in DM patients, thereby reducing the risk of DM-associated complications [93].

## 6 MECHANISMS OF ANTI-DIABETIC PROPERTIES OF NANO-CURCUMIN

The antioxidant and anti-inflammatory properties of curcumin are the key mechanisms behind its anti-diabetic effects [57]. Chronic inflammation and insulin resistance are hallmarks of DM, often resulting from the overproduction of proinflammatory cytokines by infiltrating monocytes in adipose tissue. Additionally, diabetic neuropathy, poor blood glucose control, and immune system dysfunction increase infection susceptibility [94-96]. Oxidative stress and inflammation play pivotal roles in the pathogenesis of diabetes [97]. Reactive oxygen species (ROS) activate the C-Jun NH2-terminal kinase (JNK) pathway, leading to the translocation of pancreatic and duodenal homeobox 1 (PDX-1), which disrupts β-cell function [98]. Furthermore, the accumulation of ROS and the reduced activity of antioxidant enzymes contribute to hyperglycemia-induced apoptosis, thereby exacerbating the progression of the disease [99]. Hyperlipidemia increases oxidative stress by impairing mitochondrial function and beta-oxidation [100]. This leads to a cascade of molecular events that contribute to β-cell dysfunction and inadequate insulin secretion, resulting in sustained hyperglycemia and the progression of diabetes. Fig. (**2**) illustrates the intricate mechanisms underlying diabetes-associated oxidative stress and the key molecular players involved in this pathway.

Generally, the supplementation of nCUR significantly reduced serum FBS, insulin resistance, TG, total cholesterol, and LDL-C, and also increased HDL-C and insulin levels [56, 81, 83, 84]. The function of beta cells was increased, resulting in reduced beta cell death and lower levels of inflammatory cytokines (IL-2, IL-4, IL-5, IL-6, IL-10, IL-13; IL-1α, IL-1β; IL-12p70; IFN-α, TNF-α) in the pancreatic tissue of nCUR-treated animal models [77, 82]. The nCUR supplement has also been shown to reduce ROS-induced DNA damage [77]. It suggests that anti-inflammatory effects, improvement of the lipid profile, and beta-cell health are possible mechanisms of nCUR-mediated anti-diabetic effects. Suppression of the immune regulatory pathway (NF-κB) has been reported in nCUR-treated animals [80]. Enzyme levels, such as AST, ALT, and ALP, have been reduced after nCUR treatments [80, 81, 84]. The kidney function has improved, and DM-associated kidney lesions, as well as the expression of IL-1β, NLRP3, NF-κB, casp3, and MAPK8, have been reduced. The hyperactivation of p53 and p38 MAPK has been significantly suppressed. It has been reported that nCUR, when combined with insulin, improves kidney health through the p38/p53 signaling axis [85] (Fig. **3**).

The molecular players associated with DM, which are regulated by curcumin, have been previously reported (Fig. **4**). However, a concise molecular mechanism of the anti-diabetic property of nCUR has to be elucidated.

The study's limitation: The manuscript's aim was restricted to the anti-diabetic property of nCUR only. It does not discuss the formulations, developments, evaluations, and safety aspects of nCUR.

## CONCLUSION

Due to its promising preclinical and clinical findings, nCUR has gained attention for its potential to manage DM-associated health complications. However, the molecular mechanisms underlying the therapeutic effects of nCUR are still not fully understood. This knowledge gap limits its application and potential for clinical use. Although preclinical studies have shown promising results, the clinical application of nCUR for DM and its complications remains underexplored. Several clinical trials suggest potential benefits; however, these studies are often limited in terms of scale, duration, and diversity of the populations studied. The safety, efficacy, and long-term effects of nCUR in humans remain unclear. Therefore, further detailed studies in different populations, focusing on various aspects of clinical importance, safety, and toxicity, are necessary to ensure the applicability of the nCUR. The universal guidelines for the wise use of nanomaterials and nanomedicine are needed.

## USE OF AI DECLARATION

The manuscript has been improved with the support of Grammarly.com. The content is not created using AI tools.

## AUTHORS’ CONTRIBUTIONS

The authors confirm their contribution to the paper as follows: B.S.S.: Conceptualization; investigation; writing-original draft; methodology; validation; writing-review and editing; formal analysis; project administration; data curation. K.I.: Data curation; writing-original draft. C.C.: Conceptualization; funding acquisition; writing-review and editing; resources. P.K.: Conceptualization; writing-original draft; writing-review and editing; project administration; formal analysis; data curation.

## Figures and Tables

**Fig. (1):**
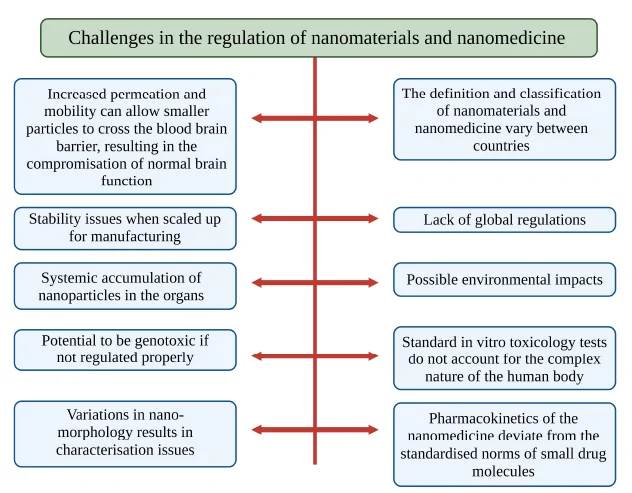
The major challenges associated with the regulation of nanomaterials and nanomedicine (Recreated based on Foulkes *et al.* [62]).

**Fig. (2):**
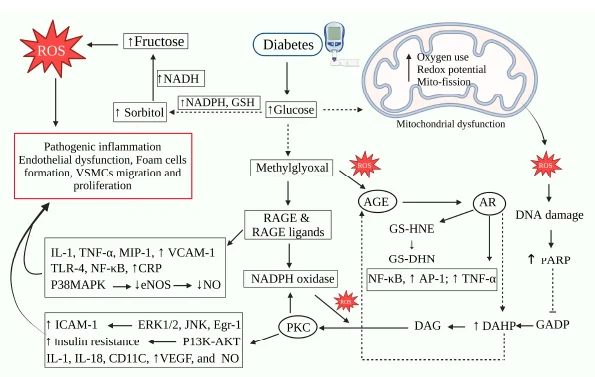
Hyperglycemia induces excessive production of reactive oxygen species (ROS) in mitochondria, which leads to significant nuclear DNA damage. This triggers the activation of nuclear poly(ADP-ribose) polymerase (PARP), which, in turn, inhibits glyceraldehyde-3-phosphate dehydrogenase (GAPDH) activity. As a result, early glycolytic intermediates are diverted into pathogenic signaling pathways, such as the polyol pathway, protein kinase C (PKC) activation, and the formation of advanced glycation end-products (AGEs). These pathways further amplify ROS production and promote inflammatory processes, thereby accelerating the progression of diabetic complications. NADH: Nicotinamide adenine dinucleotide hydrogen; NADPH: Nicotinamide adenine dinucleotide phosphate; GSH: Glutathione; RAGE: Receptor for advanced glycation end products; NF-κB: Nuclear factor kappa B; AP-1: Activating protein-1; TNF-α: Tumor necrosis factor-alpha; GADP: Glyceraldehyde 3-phosphate; DAHP: 3-Deoxy-D-arabinoheptulosonate 7-phosphate; DAG: Diacylglycerol; IL: Interleukin; MIP-1: Macrophage inflammatory protein-1; VCAM-1: Vascular cell adhesion protein 1; TLR-4: Toll-like receptor 4; CRP: C-Reactive protein; P38MAPK: p38 mitogen-activated protein kinases; eNOS: Endothelial nitric oxide synthase; NO: Nitric oxide; ICAM-1: Intercellular adhesion molecule 1; ERK1/2: Extracellular signal-regulated kinases 1 and 2; JNK: c-Jun N-terminal Kinase; Egr-1: Early growth-response gene product; P13K-AKT: Phosphoinositide 3-kinase/protein kinase B; CD11C: Integrin αX subunit; VEGF: Vascular endothelial growth factor; GS-HNE: Glutathionyl-4-hydroxy-2(E)-nonenal; GS-DHN: glutathionyl-1,4-dihydroxynonene; VSMCs: Vascular smooth muscle cells (Recreated based on Lu *et al.* [57]).

**Fig. (3):**
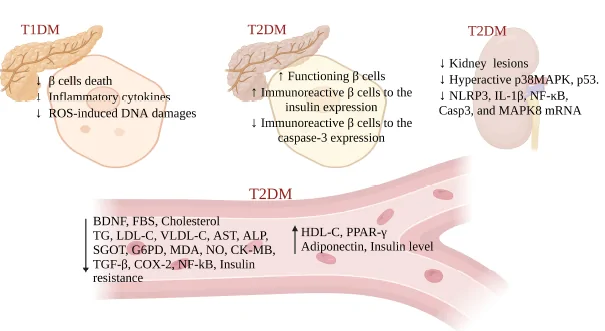
nCUR-mediated alleviation of DM-associated complications in experimental animals. The changes have been observed in the beta cells of the pancreas islets and serum parameters. nCUR treatment improved the improvement of inflammation and apoptotic pathways in the kidney. ROS: Reactive oxygen species; p38MAPK: p38 mitogen-activated protein kinases; NLRP3: NOD-like receptor protein 3; IL-1β: Interleukin-1 beta; NF-κB: Nuclear factor kappa B; Casp3: Caspase-3; MAPK8: Mitogen-activated protein kinase 8; BDNF: Brain-derived neurotrophic factor; FBS: Fasting blood sugar; TG: Triglycerides; HDL-C: High-density lipoprotein-Cholesterol; LDL-C: Low-density lipoprotein-Cholesterol; VLDL-C: Very low-density lipoprotein-Cholesterol; AST: Aspartate aminotransferase; ALP: Alkaline phosphatase; SGOT: Serum glutamate oxaloacetic transaminase; G6PD: Glucose-6-phosphate dehydrogenase; MDA: Malondialdehyde; NO: Nitric oxide; CK-MB: Creatine kinase-isoenzyme; TGF-β: Transforming growth factor-betta; COX-2: Cyclooxygenase-2; PPAR-γ: Peroxisome proliferator-activated receptors.

**Fig. (4):**
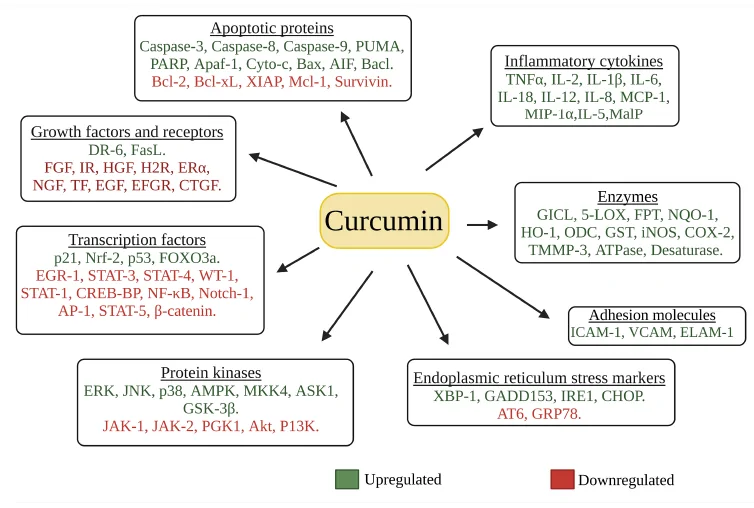
Curcumin targets several molecular players in DM and has regulated several signaling molecules by up- or down-regulating them (Recreated based on Lu *et al.* [57]).

**Table 1:** The common medications to manage diabetes.

**Drug**	**Mechanism of Action**	**Benefits**	**Limitations**	**References**
Insulin	Promoting glucose uptake into muscle and fat cells by activating insulin receptors on cell surfaces. Inhibiting hepatic glucose production.	Effective glycemic control. Improvement in lipid profile. Cardioprotective effects.	Hypoglycemia. Weight gain. Need for regular monitoring.	[16, 17]
Biguanides (Metformin)	Reduces hepatic glucose production and increases insulin sensitivity.	Considered a first-line therapy due to its effectiveness, safety profile, and affordability. Low risk of hypoglycemia. It is generally weight-neutral and may even lead to modest weight loss.	Gastrointestinal side effects (Nausea, diarrhea, abdominal discomfort, and bloating). Contraindicated in severe renal or liver impairment. Risk of lactic acidosis. Some patients are allergic to metformin.	[15]
Sulfonylureas (Glimepiride)	Promotes insulin secretion from pancreatic β-cells.	Effective in lowering blood glucose and affordable.	Risk of hypoglycemia. Weight gain. Not suitable for patients with renal or liver dysfunction.	[14]
Meglitinides (Nateglinide)		Effective insulin secretion stimulation. Alternative for sulfonylurea-intolerant patients.	Risk of hypoglycemia. Weight gain. Multiple daily doses are required. Less durable glucose control.	[14]
Thiazolidinediones (Pioglitazone)	Activates peroxisome proliferator-activated receptors-γ to improve insulin sensitivity.	Effective in lowering blood glucose. No hypoglycemia risk.	Weight gain. Fluid retention. Increased risk of heart failure. Potential bone fractures. Bladder cancer risk.	[14]
Dipeptidyl peptidase-4 (DPP-4) inhibitors (Alogliptin, Linagliptin, Saxagliptin, Sitagliptin)	Inhibits DPP-4 enzyme, prolonging active glucagon-like peptide-1 and gastric inhibitory polypeptide levels.	Weight-neutral. Low risk of hypoglycemia. Well-tolerated.	Potential increased risk of heart failure. Joint pain. Pancreatitis. Relatively expensive.	[14]
Glucagon-like peptide-1 (GLP-1) receptor agonists (Dulaglutide, Exenatide, Liraglutide, Semaglutide, Tirzepatide)	Mimic GLP-1 effects: enhance insulin secretion, inhibit glucagon release, slow gastric emptying.	Weight loss. Cardiovascular benefits. Low risk of hypoglycemia.	Gastrointestinal side effects. Potential risk of pancreatitis. Contraindicated in patients with a personal or family history of medullary thyroid carcinoma or multiple endocrine neoplasia type 2.	[15]
Sodium-glucose cotransporter-2 (SGLT2) inhibitors (Canagliflozin, Dapagliflozin, Empagliflozin, Ertugliflozin)	Inhibit SGLT2 in the kidneys, reducing glucose reabsorption and increasing urinary glucose excretion.	Weight loss. Cardiovascular and renal protection. Low risk of hypoglycemia.	Urinary tract and genital infections. Euglycemic diabetic ketoacidosis. Risk of lower limb amputations. Bone fractures. Acute kidney injury.	[14]
Alpha-glucosidase inhibitors (AGIs) (Acarbose, Miglitol)	AGIs inhibit alpha-glucosidase enzymes, which slow down the breakdown of carbohydrates. AGIs delay carbohydrate digestion and glucose absorption.	Control of postprandial blood glucose. Low risk of hypoglycemia. Weight-neutral. Affordable.	Gastrointestinal side effects. Modest blood glucose reduction. Not suitable for patients with certain gastrointestinal conditions, such as inflammatory bowel disease.	[15]

**Table 2:** Various nano-formulations of nCUR and their main benefits.

**S. No.**	**Nano-formulations**	**Major Advantages**
1	Liposomes	Enhanced solubility and stability
2	Nanomicelles	Improved oral bioavailability
3	Solid Lipid Nanoparticles	Controlled release and long-term stability
4	Polymeric Nanoparticles	Controlled release and improved bioavailability
5	Nanogels	Sustained release and enhanced stability
6	Niosomes	Improved solubility and stability
7	Magnetic Nanoparticles	Targeted delivery *via* magnetic field
8	Gold Nanoparticles	Biocompatibility and functionalization ease

**Table 3:** Results of preclinical studies on the effects of nano-curcumin on diabetes mellitus.

**Model**	**Interventions**	**Formulation**	**Dose**	**Results**	**References**
STZ and Nicotinamide-induced T2DM rats	nCUR Or Free curcumin	Nano-micelle	100 or 200 mg/kg BW/day for 28 days	↓ Insulin resistance ↓ Serum levels of FBS, Apelin, cholesterol, TG, LDL, and VLDL. ↑ HDL level.	Shamsi-Goushki *et al.* [56]
STZ-induced T1DM rat model	Nano- encapsulated curcumin	Polylactide-co-glycolide-based nanoencapsulate	10 or 50 mg/ kg BW before STZ treatment	↓ Glucose level ↓ Death of pancreatic beta cells ↓ Inflammation ↓ 8-oxo-20-deoxyguanosine	Ganugula *et al.* [77]
STZ-induced male T2DM Sprague-Dawley rats	Nano- encapsulated curcumin	Poly-Poly (ethylene glycol) methacrylate-2-(Dimethylamino)ethyl methacrylate-micro-arc oxidation-based nanoencapsulate	4 mg/kg BW/twice	↑ IL-1β, Cx43 and P2Y12 expression ↓ p-Akt level in the DRG of DM rats 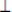 SGCs activation ↓ CGRP expression in the DRG neurons.	Jia *et al.* [78]
STZ-induced T2DM rats	nCUR or AGE suspension	Nano-curcumin sodium salt	300 or 500 mg/kg BW	AGE group: ↓ Glucose, CK-MB, and LDH activities ↓ RAGE expression. nCUR group: ↓ triglycerides, CK- MB, LDH, and AST. Mn-SOD mRNA was highly expressed in the heart tissue of nCUR and AGE-treated DM rats. Improvement of the structural integrity of the myocytes.	Abdel-Mageid *et al.* [79]
STZ-induced T2DM rats	CUR-PLA-PEG (Or) Free curcumin	polylactide-poly (ethylene glycol) copolymer nanoparticles	20 mg CUR/kg BW	↓ Liver inflammation ↓ Oxidative stress ↓ Inflammatory cytokines	El-Naggar *et al.* [80]
STZ-induced DM rats	Free curcumin or nano-micelle curcumin	Nano-micelle	40 and 80 mg/kg of feed/ day for 42 days	Improved the glucose, lipid profile, and liver enzymes.	Dadgar *et al.* [81]
STZ-induced male T2DM Sprague-Dawley rats	nCUR or Free curcumin	Chitosan-based nano formulation	100 mg/kg BW/day for 1 month	↓ Blood glucose ↑ Insulin ↑ Immunoreactive beta cells to the insulin expression ↓ Immunoreactive beta cells to the caspase-3 expression	Metawea *et al.* [82]
STZ-induced T2DM Wistar rats	nCUR Or Free curcumin	Nano-micelle	100 and 200 mg/kg BW/day for 28 days	↑ Serum BDNF ↓ FBS	Shamsi-Goushki *et al.* [83]
STZ-induced T2DM Balb/C Mice	SCCPN	Chitosan-based- nano micelles	100 mg/kg BW/day for 21 days	↓ FBS, LDL-C, ALP, G6PD, SGOT, TG ↓Total cholesterol ↑ HDL-C	Rahaman *et al.* [84]
STZ-induced male T2DM Sprague-Dawley rats	Free curcumin Or nCUR Or nCUR + long-acting insulin (LAI; Lantus®)	Poly(lactic-co-glycolic) acid-gambogic acid-based nano formulation	40 mg free curcumin/kg BW/day. 20 mg nCUR/kg BW/day. 20 mg nCUR + 2 IU Lantus^®^ for 10 weeks	nCUR and nCUR+ LAI suppressed DM-associated kidney lesions. ↓ P38 and P53 activation ↓ Expression of renal NLRP3, IL-1β, NF-ĸB, Casp3, and MAPK8 mRNA.	Ganugula *et al.* [85]
STZ-induced T2DM Wistar rats	nCUR solution	nCUR encapsulated in fenugreek mucilage	0.5 ml for 4 weeks	↓ FBS	Ferradj *et al.* [86]
Dexamethasone-induced diabetic rats	nCUR Or Free curcumin	Curcumin-loaded chitosan NPs	100 mg/kg for 14 days	↑ BW ↓ Insulin resistance, FBS, AST, ALT, cholesterol, TG, LDL. ↓ MDA in pancreatic and liver tissues. ↑HDL, albumin, GSH, SOD and GLUT4 levels	Hamed *et al.* [61]
Alloxan-induced diabetic Swiss albino.	nCUR	Polymer poly- lactide-co-glycolide-based nano-formulation	12.5 or 25 mg/kg of BW	Improved the blood glucose and insulin levels	Dey *et al.* [87]

**Note: ** ↑: Increased; ↓: Decreased; 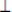: **Abbreviations: **Inhibited; STZ: Streptozotocin; DM: Diabetes mellitus; T1DM: Type 1 DM; T2DM: Type 2 DM; NPs: Nanoparticles; BW: Body weight; p-Akt: Phosphorylated-Akt; DRG: Dorsal root ganglia; Cx43: Connexin43; P2Y12: Purinergic 2 (P2) Y12 receptor; CGRP: Anti-calcitonin gene-related peptide; AGE: Aged garlic extract; CK-MB: Creatine kinase-isoenzyme; LDH: Lactate dehydrogenase; TG: Triglycerides; AST: Aspartate aminotransferase; Mn-SOD: Manganese-superoxide dismutase; RAGE: Glycation end products gene; CUR-PLA-PEG: Curcumin-loaded polylactide-poly (ethylene glycol) copolymer nanoparticles; FBS: Fasting blood sugar; LDL: Low-density lipoprotein; VLDL: Very-low density lipoprotein; HDL: High density lipoprotein; LDL-C: LDL-cholesterol; HCL-C: HDL-cholesterol; BDNF: Brain-derived neurotrophic factor; SCCPN: Succinyl Curcumin Conjugated Chitosan Polymer-Prodrug Nano micelles; ALP: Alkaline phosphatase; G6PD: Glucose-6-phosphate dehydrogenase; SGOT: Serum glutamate oxaloacetic transaminase; IU: International units; IL-1β: Interlukin-1β; NLRP3: NOD-like receptor protein 3; NF-ĸB: Nuclear factor kappa-light-chain-enhancer of activated B cells; Casp3: Caspase 3; MAPK8: Mitogen-activated protein kinase 8; AST: Aspartate transaminase; ALT: Alanine transaminase; GSH: Glutathione; SOD: Superoxide dismutase; GLUT4: Glucose transporter type 4.

## LIST OF ABBREVIATIONS

DNP: Diabetic Neuropathic Pain
SGCs: Satellite Glial Cells
SGLT2: Sodium-glucose Cotransporter-2
GLP-1: Glucagon-like Peptide-1
nCUR: Nano-curcumin
DM: Diabetes Mellitus
